# Defining the V5/MT neuronal pool for perceptual decisions in a visual stereo-motion task

**DOI:** 10.1098/rstb.2015.0260

**Published:** 2016-06-19

**Authors:** Kristine Krug, Tamara L. Curnow, Andrew J. Parker

**Affiliations:** Department of Physiology, Anatomy and Genetics, University of Oxford, Parks Road, Oxford OX1 3PT, UK

**Keywords:** Rhesus monkey, primate, visual cortex, extrastriate, binocular disparity

## Abstract

In the primate visual cortex, neurons signal differences in the appearance of objects with high precision. However, not all activated neurons contribute directly to perception. We defined the perceptual pool in extrastriate visual area V5/MT for a stereo-motion task, based on trial-by-trial co-variation between perceptual decisions and neuronal firing (choice probability (CP)). Macaque monkeys were trained to discriminate the direction of rotation of a cylinder, using the binocular depth between the moving dots that form its front and rear surfaces. We manipulated the activity of single neurons trial-to-trial by introducing task-irrelevant stimulus changes: dot motion in cylinders was aligned with neuronal preference on only half the trials, so that neurons were strongly activated with high firing rates on some trials and considerably less activated on others. We show that single neurons maintain high neurometric sensitivity for binocular depth in the face of substantial changes in firing rate. CP was correlated with neurometric sensitivity, not level of activation. In contrast, for individual neurons, the correlation between perceptual choice and neuronal activity may be fundamentally different when responding to different stimulus versions. Therefore, neuronal pools supporting sensory discrimination must be structured flexibly and independently for each stimulus configuration to be discriminated.

This article is part of the themed issue ‘Vision in our three-dimensional world'.

## 1. Introduction

Electrical stimulation of small groups of visual cortical neurons alters decisions about the perceptual appearance of visual stimuli in a predictable fashion [1,2]. This kind of experiment shows that, within the cortex, perceptual decisions are driven by the level of electrical activity within specific groups of neurons relevant for a given stimulus and task [3]. Such studies suggest that those neurons that are strongly activated by a specific stimulus and show changes in their activity (i.e. neuronal tuning) for the stimulus parameters along which a stimulus is to be discriminated will contribute to its percept. But an external stimulus excites many cortical neurons with diverse receptive field (RF) properties. When a task requires a decision about a particular perceptual attribute of the stimulus (such as its direction of motion), only a fraction of the visually stimulated neurons carry measurable perceptual signals [4–16]. This poses the question of which neurons in the population contribute to a particular decision and how a selected group of neurons is recruited to the decision pool.

Computational models provide useful insight into the principles that a neuronal decision system might follow, but experimental study is required to resolve the question of which neurons contribute to the perceptual decision [17–21]. In this paper, we aim to identify common properties of cortical neurons that contribute to the perceptual decision. In particular, we quantify the perceptual contribution of neurons that are activated by a non-preferred external stimulus (that is, a stimulus that does not excite the cell optimally). Much early work in this area examined the discrimination of movement direction between patterns of randomly positioned dots moving in opposite directions [4,5]. For example, to provide neuronal signals for supporting a discrimination between leftwards and rightwards motion, the nervous system might assemble two pools of neurons, one whose members are primarily sensitive to leftwards motion and the other primarily sensitive to rightwards movement, and then ‘read-out’ a discrimination signal by taking the difference between the activities of the two pools. It seems initially to be self-evident that neurons optimally excited by leftwards motion should be incorporated for ‘read-out’ within the pool signalling leftward motion. Even the earliest studies pointed out that this view is problematic: a neuron with a preferred direction of motion some 20° off-axis from exactly leftwards may, in practice, be superior at discriminating leftwards versus rightwards movement than any of the neurons that fire maximally when the stimulus motion is exactly leftwards [5,22,23].

Neuronal models of perceptual decision-making aim to build a quantitative account of the link between the firing of populations of single neurons and the behavioural choices to external stimuli [24,25]. Two measures of neuronal performance are widely used in assessing the contribution of single neurons to perceptual decisions [24]. First, neuronal sensitivity measures the ability of the neuron to provide reliable signals about changes in the stimulus; thus, sensitivity is an indicator of the suitability, usefulness or potential contribution of the neuron for the required task. Second, choice probability (CP) measures whether the neuron's firing is associated, on a trial-by-trial basis, with the reported perceptual decision; it is therefore potentially an indicator that signals from the recorded neuron may be read-out or that these signals are otherwise associated with the task (for example, by receiving incoming neural activity from a shared source). An important empirical result is that, for neurons that are well matched to the task, these two measures are also correlated in a sensible way. Neurons with high neurometric sensitivity tend also to have higher choice probabilities; in other words, more sensitive neurons have generally a stronger association with the decision [4,5,8,10,14,26]. These observations were predominantly conducted with stimuli that are close to optimal in respect of generating a response in the recorded neurons.

When a neuron does not respond to visual stimuli or cannot discriminate between them, the neuron will also not carry a CP [5]. Reducing the information a neuron can provide, for example by rotating the motion axis for the discrimination task away from a neuron's direction preference reduces neuronal sensitivity and CP [27], but implicitly also reduces the neuron's firing rate. Two studies have systematically probed this association between task sensitivity and choice probabilities in visual motion tasks across a wider neuronal population, exploring the role of neurons that are not well matched to visual stimulus and task. Purushothoman and Bradley [10] examined neuronal responses of direction selective neurons in cortical area V5/MT whilst monkeys performed a fine-scale discrimination of motion direction [10]. The motion stimuli and discrimination task remained the same while neurons with different direction selectivity and sensitivity were tested to simulate population pooling. Behavioural thresholds for the discrimination task were on the order of 1–2°, whilst very few neurons showed performance approaching this level. Nonetheless, the population of neurons showed a small but significant CP, with the more sensitive neurons showing larger choice probabilities, as found earlier [4,5,26]. Consequently, Purushothoman and Bradley [10] argued that the neuronal pool responsible for the perceptual decision must be large and diffuse, incorporating neurons that only respond weakly to the stimulus, although the set of neurons with high sensitivity and large choice probabilities makes a stronger contribution [10]. However, due to the experimental design, task sensitivity was inextricably linked to neuronal selectivity for direction of motion ([10] see their fig. 3*b*) and therefore, the relationship of level of activation and neurometric threshold to CP could not be determined independently.

Bosking and Maunsell [28] examined a reaction-time variant of the Newsome *et al.* [29] motion task, based on the detection of coherent movement in a field of dots [28,29]. They also found that the neuronal pool responsible for the decision was broad in relation to neuronal tuning. They used a measure similar to CP to assess the association of the neuron with the trial-by-trial variations in the animal's behavioural performance when detecting the onset of a coherent motion stimulus [30]. This measure is called ‘detect probability’ and it characterizes the link between the animal's decision and the neuronal activity on trials when the stimulus content clearly indicates the correct outcome of the decision but the animal nonetheless makes mistakes. Strictly, this differs from CP, as used in this paper, in that measurement of CP applies only to trials on which a truly ambiguous stimulus is presented, such that the animal's decision must be rewarded at random at the end of the trial and there is no visual content within the stimulus to determine the discrimination one way or the other. Bosking and Maunsell found that detection probability was highest near the peak of the direction tuning curve, showing a steady decline away from this peak [28]. They noted a negative correlation between behavioural choice and the firing of neurons whose stimulus preference was directly opposite to the choice indicated. Again, changing the motion axis of the visual stimulus relative to a neuron's preferred axis of motion must systematically affect both factors: neuronal selectivity for the stimulus, which relates to the level of response to the visual stimulus, and neurometric sensitivity, which is a task-specific property of the neuron.

Both the above-mentioned studies manipulated the visual stimulus dimension crucial to the judgement. It appears to be a consequence of such an experimental framework that both the neuronal tuning preference and neurometric threshold are linked to each other as well as to CP ([10], see figs. 3*b* and 4*e*; [19], see figs. 4*c* and 6*a*). Therefore, in defining which neurons belong to the perceptual pool, the role of task sensitivity of a neuron could not be separated from its selectivity for the visual stimulus, as in this paradigm both of these factors are linked for a single neuron to its absolute level of firing rate. Again in this paradigm, the use of choice or detect probabilities as measures of perceptual decision signals were also confounded by the use of stimuli that contained directional motion signals [31]. As a consequence, these previous studies can accommodate their findings within a simple framework, in which sensory neurons are included in one of the two pools, each pool favouring one of the two alternatives in the decision task.

**Figure 1.**
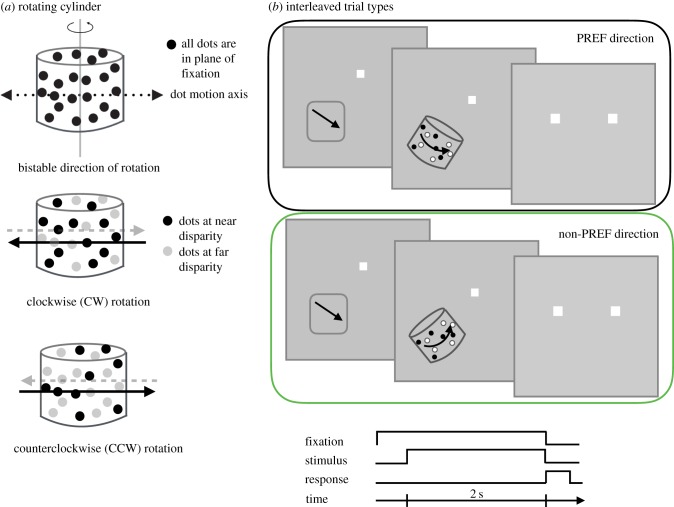
(*a*) Visual stimuli were defined by two sets of oppositely moving dots, each set moving with a sinusoidal velocity profile along the axis of dot motion, to create the impression of a solid cylindrical form rotating around a central cylinder axis. A cylinder with a vertical cylinder axis (defined as −90°) has horizontal dot motion. Binocular disparity could be added to the dots so that the cylinder's direction of rotation around its axis was unambiguous. (*b*) Following basic measurements of the visual receptive field (RF) properties (grey rectangle with arrow), trials began with a blank screen and a fixation marker. After the monkey looked at the marker, a cylinder was placed for 2 s in the RF of a direction- and disparity-selective neuron with one direction of dot motion aligned with the preferred (PREF) direction of motion (arrow direction) whilst the monkey held fixation on the marker. For these trials, the axis of cylinder rotation was optimal in that it included dot motion that was in the PREF direction for the neuron's RF. At the end of the trial, the cylinder and fixation marker disappeared, two choice targets appeared and the monkey indicated the direction of rotation around the cylinder axis by choosing the left (CW rotation) or the right choice target (CCW rotation). The animal was rewarded for a correct choice on unambiguous trials (when horizontal disparity defined the cylinder's direction of rotation) and on a randomly selected 50% of ambiguous trials. Trials with the optimal cylinder axis were interleaved with trials during which the cylinder axis was sub-optimal in that the dot motion was not aligned to the direction preference of the neuron (here, about 80° away from optimal). The animal responded to the same choice targets to indicate the direction of rotation and the same set of disparity values was used. The animals' performance with interleaved cylinder orientations was carefully monitored throughout.

Our study challenges this simple account by arranging task-irrelevant changes in the stimulus, which alter the activation profile of sensory neurons from trial to trial. Our paradigm uses a rotating cylinder defined by the visual motion and binocular disparity of a set of dots presented on a computer display (figure 1*a*). The monkeys' task was to judge whether the direction of rotation of the cylinder was either clockwise (CW) or counterclockwise (CCW). This judgement depends on the binocular disparity between the oppositely moving sets of dots that define the cylinder. Each neuron was probed systematically with two differently oriented cylinders, such that only one of the cylinder stimuli contained dot motion in the preferred direction of the recorded neuron (figure 1*b*). These dot motion changes in the stimulus were irrelevant for the task, but the cortical machinery must nonetheless extract a relevant decision variable to drive the discrimination task in the face of this irrelevant variation. Thus, we made simultaneous measurements of the neurometric sensitivity and CP of single V5/MT neurons, under conditions in which an irrelevant change in the stimulus results in it being either nearly optimally matched to the neuron's RF or results in distinctly lower levels of activation. This manipulation separated the factor of neuronal selectivity for the stimulus from the task-specific factor of neurometric sensitivity.

Consequently, we are able to show that task sensitivity, not visual selectivity for the stimulus, determines a neuron's contribution to perceptual decisions. We also find that the neuronal pool is potentially broad with respect to stimulus selectivity. However, while a neuron might be task sensitive across a range of irrelevant stimulus changes, our results show that it might not carry perceptually relevant signals for all these variants. Thus, we propose that neuronal pools contributing to perceptual decisions are specific for a particular stimulus configuration and much smaller than the pool of task-sensitive neurons implies.

## 2. Methods

### (a) Animals

A detailed description of techniques, training and procedures is given elsewhere [6]. All procedures underwent local ethical review at Oxford University and complied with the United Kingdom Home Office regulations on animal experimentation. In brief, two adult male macaque monkeys (*Macaca mulatta*) were trained to fixate and make visual discriminations for fluid rewards while viewing binocular stimuli via a mirror stereoscope. Their skulls were implanted with a central head post and a cranial chamber over occipital cortex under general anaesthesia. During all experiments, the horizontal and vertical positions of both eyes were measured using a magnetic scleral search coil system (CNC Engineering, Seattle, WA) [6,7].

### (b) Visual stimuli

The main stimulus was an orthographic projection onto a computer screen of a pattern of dots placed at random locations on the surface of a transparent three-dimensional cylinder rotating about its principal axis [32]. Individual dots were presented on a mid-grey background (42 cd m^−2^) and were randomly assigned to be black or white (Michelson contrast 99%). The dot size was normally 0.2°× 0.2° and the dot density was 25%. Individual dots were eliminated on each frame of the display with a probability of 0.02 and new dots were plotted at a new random location. The viewing distance was 85–89 cm. Further details of the computer and Eizo display system are given elsewhere [6]. In describing the cylinder stimulus, we refer to two axes (figure 1*a*): the principal axis of the cylinder about which it rotates (cylinder axis) and the axis of dot motion at 90° with respect to the cylinder axis. In our experiments, the axis of dot motion is chosen such that on half the trials, it is aligned with the RF's preferred direction for moving dot patterns. On the other half of the trials, the dot motion axis does not align with the preferred direction (figure 1*b*).

Cylinder stimuli are distinguished, not only by axis orientation but also according to their direction of rotation about this axis. This is defined by the combination of direction of dot movement and binocular disparity (figure 1*a*). For a cylinder with a vertical axis, a positive cylinder disparity applies a crossed horizontal disparity (near depth) to rightward moving dots and an uncrossed horizontal disparity (far depth) to leftward moving dots. Overall, this defines a CCW rotation (as viewed from above). The opposite combination of disparity and motion direction results in a CW rotation. When the cylinder axis was not vertical, CW rotation was defined as any cylinder for which the dots on the front surface were moving leftwards within the range of ±60° of the horizontal axis of dot motion. The magnitude of the cylinder disparity (defined as the horizontal binocular disparity between the principal axis of the cylinder and the dots at the point on the cylindrical surface that is nearest to the observer) controls the degree of ambiguity in the direction of rotation. For large cylinder disparities, the direction of rotation is unambiguous. As the cylinder disparities approach zero, observers perceive, with increasing frequency, the rotation opposite to that defined by the sign of cylinder disparity. When all the dots are at zero cylinder disparity, the direction of rotation is bistable. In human observers, the percept of a bistable cylinder persists in one state for more than 30 s on average [33].

### (c) Behavioural task

Animals were fluid controlled when they carried out these experiments. Once the monkey acquired the fixation marker, a cylinder appeared on the screen, positioned over the RF of the recorded neuron. Trials were aborted if the eyes moved outside a fixation window (total width 1.0°–1.6°) during the 2 s stimulus duration. After the stimulus presentation, both the fixation marker and the stimulus were extinguished and two targets were shown. Regardless of the stimulus, the targets were always positioned in the same location relative to the former position of the fixation marker: one to the left and one to the right (figure 1*b*). The monkey indicated the perceived direction of rotation by making a saccade to one of the choice targets. A correct choice was rewarded with fluid; an incorrect choice was followed by a chequered pattern for a brief time before the next trial started. A correct choice was defined as a saccade to the choice target consistent with the direction of rotation specified by the cylinder disparity: left target for CW rotation and right target for CCW rotation. For the zero-disparity cylinder, the monkey was rewarded on half of the trials, determined at random. The other half of trials was treated like genuine ‘incorrect trials' for non-zero disparity cylinders.

### (d) Combined neurophysiology and psychophysics

Action potentials were recorded via extra-cellular parylene-coated tungsten micro-electrodes (0.6–1.2 MΩ impedance at 1 kHz; Micro Probe Inc., Gaithersburg, MD), which were inserted transdurally via a guide tube before each recording session. After isolating a single unit, the preferred direction of motion of its RF was determined qualitatively using a circular patch of moving random dots at zero disparity. The minimum response field (MRF) was mapped using a rectangular patch of dots moving in the preferred direction. The speed of dot motion was qualitatively matched to the preference of the RF.

Motion-direction tuning functions were then obtained using a circular patch of dots covering the MRF. We measured the response to patches of dots, with the patch centred on the RF and the dots moving at a minimum of 10 different directions of motion over the 360° range. A Gaussian curve was fitted to the responses as function of direction: the mean and standard deviation were used, respectively, to estimate the direction of the preferred–null motion axis and the spread of tuning.

During data collection, the experimenter examined the on-line displays of tuning from the preliminary experiments and selected a pair of cylinder orientations, one that gave dot motion close to the preferred–null axis of the RF and the other that was distinctly away from this axis. The angle between the two selected orientations was in the range 20°–80°. The cylinders' dot motion was always within ±60° of the horizontal (see above), so the overall task (CW and CCW discrimination) would not change. Disparity tuning curves were measured for the two cylinders, whose size and location matched the MRF.

Choice probabilities (CPs) were measured only for neurons that showed tuning for the direction of cylinder rotation. Stimulus parameters were matched to the properties of each neuron under study except for the cylinder axis orientation of the stimulus not aligned with the preferred–null motion direction axis of the RF. To control the animals' behaviour during CP measurements, cylinder stimuli were presented with at least seven different disparities for each of the two axis orientations (one such that dot motion was optimally aligned with the direction preference of the neuron and one sub-optimal). The range of cylinder disparities was centred on zero. Hence, each block contained equal numbers of CW and CCW cylinders, as well as the ambiguous (zero-disparity) cylinders. In addition, equal numbers of optimally and sub-optimally oriented stimuli were presented. Stimuli were presented in pseudo-random order. The magnitude of the cylinder disparities was chosen to ensure that the animals were working near psychophysical threshold (−0.15° to 0.15°). Choice probabilities were calculated only from ambiguous (zero-disparity) trials (see below).

### (e) Data analysis

Neuronal responses collected from the same neuron but for pseudo-randomly interleaved cylinder stimuli with different axis orientations were analysed separately in all instances.

#### (i) Square root transform

The variance of neuronal firing rates increases approximately linearly with the mean firing rate [34–36]. To remove this relationship (so that least-squares regression analysis can be applied), tuning curves were fitted to the square root of the firing rates rather than the raw spike rates [37,38].

#### (ii) Tuning for direction of motion

The MRF of the neuron was probed with a patch of randomly located dots. The size and location of the patch was matched to the MRF and the dots moved coherently across this region. The neuron's response was recorded at a minimum of 10 different directions of motion over the 360° range. A Gaussian curve was fitted to the mean of the square root firing rates minimizing the sum of the squared errors. (Note that it might be more logical to fit a curve using a circular function, but the Gaussian, in general, provided a good fit and produced easily interpretable parameters). The Gaussian curve is of the form2.1
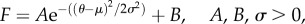
where *A*, *B*, *μ* (the mean) and *σ* (the standard deviation) are constants, *θ* is direction of motion and *F* is the square root spike rate.

The fit was determined using the Matlab (The MathWorks Inc., Natick, MA) implementation of the Nelder–Mead simplex algorithm, which sought a local minimum to the squared error [39]. A fitted curve was considered adequate if the algorithm converged on a local minimum with *A*, *B*, *σ* > 0. A sequential *F*-test [40] was used to test whether the Gaussian curve fitted to the motion-direction tuning data was a better fit than a horizontal line through the mean spike rate. A neuron was considered tuned if the modulation of spike rate by motion direction was significant at the 5% level of the *F*-distribution.

#### (iii) Assessing the preference for cylinder orientation

Choices about the orientations of the cylinders were made on-line during the experiment, so there was inevitably some uncertainty about whether the chosen orientation for the optimal cylinder resulted in dot motion that lay exactly along the preferred–null motion axis for the neuron. This uncertainty was resolved during the subsequent data analysis. A cylinder axis was considered as well matched to the RF's motion preference for a given neuron if it resulted in a dot motion that lay within one standard deviation of the mean of the Gaussian function fitted to the direction-of-motion tuning data. A cylinder axis was considered as sub-optimally aligned with the preferred–null motion axis, only if the neuron's response to the cylinder was also significantly lower than its response to the optimally oriented cylinder with zero disparity (two-tailed *t*-test, *p* < 0.05). Thus, in the direct comparisons in this paper, we can be clear that the cylinder motion axis designated as sub-optimal produced a lower neuronal response than the cylinder motion determined as optimal.

#### (iv) Tuning for direction of cylinder rotation

Tuning for the direction of cylinder rotation (CW or CCW) was measured separately for each differently oriented cylinder. Neurons were classified as selective for the direction of cylinder rotation at a particular cylinder axis orientation if there was significant modulation in the square root firing rate at the 5% level on a one-way ANOVA. The direction of rotation generating the higher response is termed the preferred (or PREF) rotation. The opposite direction is the NULL direction of rotation.

#### (v) Neurometric threshold

The neurometric threshold is the difference between stimulus levels at which an observer of the neuron's response would achieve a criterion level (84% correct in this case) in discriminating the two stimuli. In this case, the relevant discrimination requires the observer to use the neuronal response to binocular disparity to assign the direction of cylinder rotation, on the basis of the action potentials acquired over the 2 s stimulus presentation. Response distributions to two presentations of cylinders of the same absolute cylinder disparity *d* but rotating in the opposite direction were compared using signal detection analysis. The separation between the two distributions is measured by calculating the area under the receiver operating characteristic (ROC) curve [41–43]. This gives the probability of an independent observer being able to distinguish an unambiguous cylinder rotating in the neuron's PREF rotational direction from an unambiguous cylinder rotating in the NULL direction: the ‘neurometric’ probability PNM(*d*). By definition, PNM(*d*) = 1 − PNM(−*d*) and so PNM(0°) = 0.5. Neurometric probabilities were plotted against cylinder disparity and a cumulative Gaussian curve was fitted to the data. The fit was accepted if it accounted for 75% of the variance in the neurometric probabilities and also was significantly different from a horizontal line at the 5% level on a sequential *F*-test. The neurometric threshold is the standard deviation of the fitted cumulative Gaussian curve.

#### (vi) Choice probability

CP is the probability that an independent observer could predict the monkey's choice on an ambiguous trial based on the neuron's firing rate on that trial (and knowing the distribution of firing rates). Except for the analysis of time course in the last part of the paper, we calculated choice probabilities on the firing rates obtained during the entire stimulus presentation over the 2 s trial. Firing rates of zero-disparity cylinder trials were separated according to the choice made by the monkey. The separation between the two distributions can be measured using signal detection theory, by calculating the area beneath the ROC curve [4–6,41]. This area is the CP. A CP near 1 means that, with an ambiguous stimulus, the neuron nearly always fires more when the monkey chooses the neuron's PREF direction of rotation. Conversely, a CP near 0 would mean that the neuron tends to fire more for an ambiguous stimulus when the monkey's perceptual choice is the neuron's NULL direction of rotation. A CP near 0.5 indicates that the activity of the neuron is not correlated with the perceptual choice the monkey makes. We assigned the PREF rotation direction separately for optimal and sub-optimal stimuli. In a very small number of cases, the rotation preference did not match between optimal and sub-optimal cylinders. Switching the PREF sign for this small number of sub-optimal stimuli does not alter the central findings of this paper.

For a neuron's data to be included in the analysis, the requirement was at least 10 repeated presentations at each cylinder orientation with the zero-disparity, ambiguous stimulus. We included only neuronal data from blocks of trials in which the animal's behavioural performance demonstrated that it was accurately responding to the cylinder disparity. Therefore, we required that the animal scored over 90% correct at the extreme values of cylinder disparity tested, typically 0.006°–0.03°. In addition, we required that the animal's responses to the zero-disparity cylinder were unbiased. Any experiments in which the animal selected the same target on more than 80% of the zero-disparity cylinder trials were discarded. These precautions are necessary to ensure that variations in overall level of task performance do not bias the estimate of the task-specific signalling of the neuron.

We used a permutation test to measure the significance of each CP [5,44]. For the test, 10 000 ROC curves were produced by retaining both the actual spike counts observed and the relative proportion of the two choices, but randomly permuting the spike count/choice associations. The areas under the 10 000 ROC curves generated a distribution of CP values that could have arisen by chance (permutation distribution). If the observed value from the experiment lay outside of the central 95% of the values in the permutation distribution, then it was considered as significantly different from 0.5.

The quantitative analysis in this paper uses a logit transform of CP. Means were calculated by taking the mean value of the transformed data and then applying the inverse transform to return the mean to a CP scale. Thus, the mean was calculated on a less-skewed dataset than the raw choice probabilities. All other CP statistics were also calculated on the transformed data. Data reported are all inverse transformed onto the CP scale.

#### (vii) Time course of choice probability

Only neurons with significant choice probabilities were used for this analysis. For each ambiguous stimulus trial, post-stimulus time histograms were constructed (bin width 41.7 ms) and coded according to perceptual choice for cylinder rotation at the end of the trial. For the cumulative analysis, spike counts were summed across earlier bins. Choice probabilities were calculated for each bin and then averaged over all neurons that showed a significant CP for the cylinder configuration tested.

## 3. Results

Two rhesus monkeys judged the direction of rotation of a cylinder defined by the visual motion and binocular disparity of a set of dots on the computer display (figure 1*a*). The critical information for this judgment comes from the binocular disparity between the oppositely moving sets of dots that define the cylinder. While the task stayed the same (judgement of CW or CCW rotation), the monkey was presented on each trial with one of the two versions of the cylinder stimulus, which differed in the cylinder axis (figure 1*b*). One axis of rotation was always chosen to align the movement of the dots as close as possible to the preferred direction of motion of the neuron's RF, while the other axis was misaligned so that the resulting dot motion led to substantially lower rates of neuronal firing. The two versions of the cylinder were each presented with the same range of binocular disparities.

This paradigm offers a number of advantages. First, choice probabilities with a single cylinder carefully aligned to the RF are known to be large [6]. Our paradigm allows us to compare the responses to cylinders that differ only in their degree of alignment with the RF and to quantify the effect of misalignment on firing rate, neurometric sensitivities and choice probabilities of single neurons. Second, the paradigm allows the measurement of neuronal sensitivity and CP in the same neuron with a pair of stimulus sets that result in distinctly different levels of firing for that neuron. In other paradigms [10], these comparisons could only be made *between* different neurons in response to the same stimulus set. In this paradigm here, we observe the same neuron in two different states of activation. Third, our paradigm better approximates the situation in the natural world, in which decisions about a particular feature of the visual world have to be made in the context of variation in other aspects of the stimulus. In our case, the animals must respond to binocular depth in the face of variation in the direction of dot motion in the stimulus, which task-irrelevantly alters the response of the neurons.

Our aim was to acquire measures of neuronal sensitivity and CP from single neurons in cortical area V5/MT. After isolating the spiking activity of single neurons, we determined its RF and quantified the preferred–null motion axis of the neuron. In the main experiment, the two monkeys discriminated the direction of rotation of a random dot cylinder positioned over the neuron's RF. Two independent parameters were explored, interleaved in a pseudo-random order: the disparity added to the oppositely moving surfaces of the cylinder (at least seven disparities, including a zero-disparity, ambiguous cylinder) and the orientation of the cylinder (see figure 1 and §2). We analysed only neuronal data from blocks of trials for which the animal's behavioural performance demonstrated that it was accurately responding to the cylinder disparity. Interpretation of choice probabilities depends upon an accurate assignment of the neuron's preference for the direction of cylinder rotation (CW or CCW). The preferred rotation direction was assessed separately for the optimal and the sub-optimal cylinder (see §2). Neurons were classified as selective if there was significant modulation of firing rate by disparity using a one-way ANOVA (*p* < 0.05).

**Figure 2.**
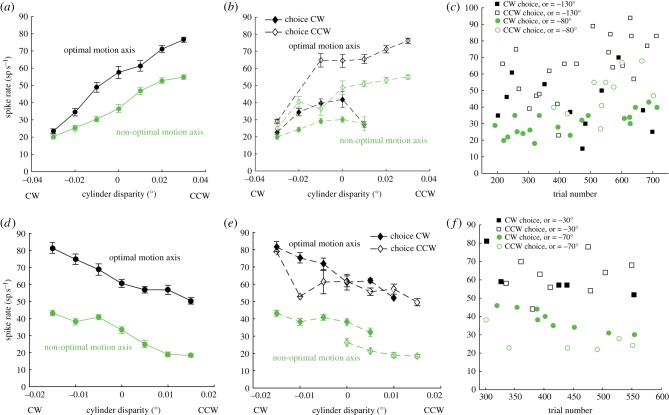
(*a*–*c*) One example of a V5/MT neuron (fle092) tuned to CCW rotation based on the binocular disparities applied to the dots forming the cylinder's front and rear surfaces. (*a*) Cylinder tuning for binocular disparity is present whether the cylinder axis is optimally aligned with the RF's preference for direction of motion (cylinder axis =−130°; black data points) or not (cylinder axis =−80°; green data points). When dot motion is not aligned with the preferred motion direction (sub-optimal cylinder axis), the neuron fires on average fewer action potentials. (*b*) For the same stimulus, choices for the preferred rotation (CCW rotation; open symbols) are preceded by higher firing rates than choices for the null rotation (CW; filled symbols). (*c*) The correlation between firing rate and subsequent perceptual choice can be seen trial-by-trial for zero disparity cylinders, whose direction of rotation is ambiguous. These data form the basis for the CP calculations. In this case, both choice probabilities were significantly different from chance (optimal cylinder CP = 0.83; sub-optimal cylinder CP = 0.91; permutation test *p* < 0.001). Green data points are results for sub-optimal cylinder stimuli and black data points are for stimuli that are optimally aligned. (*d***–***f*) Data from another example neuron (fle061) with a rotation preference for CW rotation, which showed a CP for the less well matched cylinder, which elicited significantly lower firing rates, but not for the cylinder optimized to the motion preference of the RF. Same conventions as in (*a*–*c*). (*d*) Neuron is tuned for CW rotation, both with the dot motion of the cylinder optimally aligned to the preferred direction of motion (black) and for the sub-optimal cylinder axis (green). (*e*) For the same sub-optimal cylinder stimulus, firing rates are generally higher for subsequent CW choices (green, filled symbols) than for CCW choices (green, open symbols). This is not consistently so for the optimal cylinder (black). (*f*) Trial-by-trial correlations between firing rate and perceptual choice give a significant CP for the sub-optimal cylinder (CP = 0.92; permutation test *p* < 0.01; green) but not the optimal cylinder (CP = 0.44; permutation test *p* = 0.61; black).

Figure 2 shows two examples of neurons from which we recorded (‘fle092’: figure 2*a*–*c*; ‘fle061’: figure 2*d*–*f*). Both neurons are well tuned to the binocular disparity that defines the cylinder rotation, showing monotonic relationships between binocular disparity and firing rate (figure 2*a*,*d*). In both cases, the cylinder stimulus with the dot motion that was well matched to the neuron's RF preference yielded substantially higher firing rates (black) than the cylinder with sub-optimally aligned dot motion (green). When we further separate each tuning curve according to the cylinder rotation chosen behaviourally at the end of a given trial (figure 2*b*,*e*), we find that the perceptual choice of the monkey is also related to neuronal firing. For the neuron in figure 2*a*, a cylinder with unambiguous CCW rotation is the preferred visual stimulus. A choice in favour of CCW at the end of a trial is also preceded on average by a higher firing during the trial (figure 2*b*, open symbols) than a choice of CW for the same stimulus (figure 2*b*, closed symbols). This neuronal signal related to animal's perceptual choice, measured trial-by-trial for the ambiguous, zero-disparity cylinder, is the basis for the calculations of choice probabilities below (figure 2*c*).

Figure 2*d*–*f* shows an example of an unexpected case. Here, a choice signal that correlates with the reported percept is found only for the cylinder stimulus that is not aligned to the preferred motion direction of the neuron (figure 2*e*,*f*—green symbols and lines for the non-optimal stimulus). There is no choice signal for the cylinder with dot motion in the visually preferred motion direction (figure 2*e*,*f*—black), even though the neuron is responding more strongly to that stimulus. Thus, independent of very different overall response levels to specific cylinder stimulus configurations, neurons can still show choice-related changes in firing rate.

In total, we recorded from 112 neurons that satisfied our criteria for both behavioural performance and a neuronal preference for CW or CCW rotation of the unambiguous cylinder for at least one cylinder orientation. Sixty-one of these 112 neurons (43 from Monkey F and 18 from Monkey R) were tuned to the direction of cylinder rotation at both of the tested orientations. There were also neurons for which the behavioural performance or cylinder-disparity tuning criteria were satisfied at only one of the two tested orientations. We therefore defined larger samples of neurons satisfying the inclusion criteria for at least one orientation: *Q*_pref_ (all neurons with data available from a cylinder with dot motion in the preferred–null motion axis) consisted of 93 neurons (61 from Monkey F and 32 from Monkey R) and *Q*_non-pref_ (all neurons with data available from a cylinder with dot motion not aligned with the preferred motion direction and yielding significantly lower firing rates than the optimally aligned cylinder) consisted of 68 neurons (46 from Monkey F and 22 from Monkey R).

### (a) Neurometric thresholds for cylinder disparity do not correlate with motion axis or firing rate

**Figure 3.**
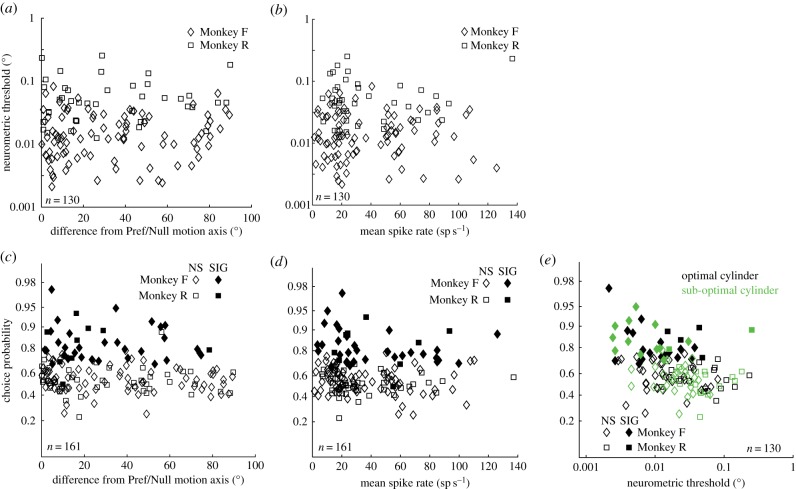
(*a*) Sensitivity of neurons to the disparity in the cylinder stimulus as a function of cylinder dot motion relative to the RF direction preference. The ordinate plots neurometric threshold in degrees (see §2). The abscissa shows the axis of movement of the dots forming the cylinder with respect to the preferred–null motion axis of the RF (determined in preliminary experiments). The data points show 130 samples recorded from area V5/MT of two monkeys (F and R) with the cylinder paradigm shown in figure 1. There was no significant correlation for either Monkey F (*r* = –0.01, *n* = 89, *p* > 0.4) or Monkey R (*r* = 0.04, *n* = 41, *p* > 0.4). (*b*) For the same data set as (*a*), neurometric threshold also did not correlate with mean firing rate generated by the zero disparity cylinder (*r =* –0.006, *n* = 130). (*c*) CPs of V5/MT neurons to zero-disparity, ambiguous cylinder stimuli as a function of cylinder dot motion axis with respect to RF preference. The ordinate shows CP for the whole pooled dataset from two monkeys fulfilling the inclusion criteria for CP measurements (*n* = 161). The abscissa shows the difference between the preferred motion direction axis for the neuron and the direction of movement of the dots forming the cylinder, as in (*a*). Filled symbols show neurons with significant CPs, open symbols show non-significant values (permutation test, *p* < 0.05). The axis scale for the ordinate provides equal steps on a logit-transformed scale for CP to facilitate estimation of differences in CP at different values. There was no significant correlation between the direction of motion in the cylinder stimulus and CP (*r* = *–*0.11, *p >* 0.15). (*d*) Same data and conventions set as in (*c*), except that the abscissa now represents mean firing rate for the zero-disparity cylinder. There was no significant correlation between CP and mean neuronal firing to zero-disparity cylinders (*r* = –0.037*, n* *=* 161*, p >* 0.4). (*e*) More sensitive neurons have bigger choice probabilities. The ordinate plots CP for zero-disparity cylinders and the abscissa plots neurometric threshold for the cylinder disparity defining rotation. Same conventions as in (*c*) and (*d*); data set fulfills the inclusion criteria for neurometric threshold and CP (*n* = 130). Green data points represent results for sub-optimal cylinder stimuli and black data points are for optimal stimuli.

The next step was to assess the sensitivity of neurons for optimally and sub-optimally oriented cylinders in this interleaved task. We computed the neurometric threshold [42] for disparity discrimination separately for cylinders whose dot motion was either optimally or sub-optimally aligned with the neuronal preference. The neurometric threshold is defined as the difference in stimulus disparities that supports a consistent level of statistical discrimination in neuronal firing (i.e. the difference between the 50 and 84% points on a cumulative Gaussian curve fitted to the neuronal discrimination data: see §2 for details). Neurometric thresholds may be calculated only for neurons with near-monotonic tuning to cylinder disparity. We could adequately define neurometric thresholds for 75 of 93 neurons in the dataset *Q*_pref_ and 55 of 68 neurons from *Q*_non-pref_. The quantitative relationship between neurometric threshold and stimulus motion with respect to the RF preferences is presented graphically in figure 3*a*. The most striking feature is the presence of neurons with low thresholds for discriminating binocular disparity, when the neuron is presented with cylinders made up of dots whose motion is as much as 70° away from the preferred–null motion axis for the neuron (figure 3*a*). This shows the separation between the task-specific measure of the neuron's responses (neurometric threshold) and unrelated visual stimulus preferences of the neuron (degrees of rotation from the preferred–null motion axis). In fact, there is no significant correlation between neurometric threshold and cylinder stimulus motion (figure 3*a*). Comparison of thresholds for the two data sets *Q*_pref_ (mean threshold 0.018°; Monkey F: 0.012°; Monkey R: 0.045°) and *Q*_non-pref_ (mean threshold 0.021°; Monkey F: 0.014°; Monkey R: 0.052°) also shows no significance (*t*-test, *p >* 0.4, for each monkey considered separately, nor for both monkeys taken together). Regardless of cylinder orientation, there was a significant difference in neurometric thresholds between the two monkeys (*p <* 0.0001, *t*-test, at both orientations), which may reflect differences in the monkeys' psychophysical performance (Monkey F: 0.008°; Monkey R: 0.029°; *p <* 0.0001, *t*-test).

We wondered whether broadly tuned neurons might maintain high neurometric sensitivity over a wide range of cylinder orientations, thus contributing to the absence of any relationship between sensitivity and the direction of dot motion in figure 3*a*. We therefore also examined the relationship between neurometric threshold and stimulus motion by expressing the direction of motion as a standardized difference based on the spread of the tuning curve (standard deviation of the best fitting Gaussian). As for the data in figure 3*a*, there was no significant correlation for either Monkey F (*r* = 0.08, *n* = 89, *p* > 0.4) or Monkey R (*r* = –0.03, *n* = 41, *p* > 0.4). Low neurometric thresholds can be identified for stimuli with dot motion that is up to 1.7 standard deviations from the preferred–null motion axis. At these values, mean neural firing is some 25% of the firing at the peak of the direction tuning curve. In summary, there was no significant correlation for either monkey between neurometric threshold for binocular disparity and the deviation in the direction of dot motion from the RF preference, expressed either in degrees or in standardized units.

The design of our study also allows a direct, within-neuron comparison. For 42 neurons, there were data fulfilling the criteria for neuronal tuning and behaviour for both orientations of cylinder with interleaved trials of the two. One cylinder had dots moving in the preferred motion direction of the neuron and the other cylinder had dots misaligned with the preferred–null motion direction axis. This allows a comparison of thresholds in the same neuron for optimally and sub-optimally aligned cylinder stimuli: mean neurometric threshold for optimal was 0.015° (Monkey F: 0.010°, Monkey R: 0.039°) and for sub-optimal was 0.018° (Monkey F: 0.012°, Monkey R: 0.054°), with a strong correlation between the two measures of neurometric threshold (Monkey F: *r =* 0.72, *n =* 30, *p <* 0.0001; Monkey R: *r =* 0.59, *n =* 12, *p <* 0.05). We found no significant difference in neurometric threshold between optimal and sub-optimal stimuli (*p >* 0.05, paired *t*-test and Wilcoxon signed-rank test, for both monkeys combined or for each separately).

In our experiments, during the performance of the task with a cylinder whose dot motion was not aligned to a neuron's preferred direction of motion, the firing rates on ambiguous trials were on average reduced to 55% (geometric mean) of the firing rates generated by an optimally aligned ambiguous cylinder. Therefore, single cortical neurons can effectively maintain high sensitivity for sensory discrimination under the challenge of alterations in the stimulus that bring about substantial changes in the neuron's firing rate. Indeed, there was no correlation between neurometric threshold and firing rate for the whole dataset (*r =* –0.006, *n* = 130) or for each monkey separately (Monkey F: *r = –*0.007, *n =* 89, Monkey R: *r =* 0.076*, n =* 41) (figure 3*b*).

The principle that neurons with low firing rates may exhibit high neuronal sensitivity has been established before [45]. However, this observation was made across different neurons with various levels of responsiveness. Our data go further in showing that individual cortical neurons succeed in preserving their sensitivity as their firing rate changes substantially in response to a task-irrelevant stimulus parameter. We explored this property further by examining variation in neuronal firing across repeated presentations of the same stimulus, estimating both the mean and variance of the spike counts across our 2 s trials. These data were summarized by the variance to mean ratio [35], which did not differ significantly between optimal and sub-optimal cylinder orientations. This indicates that the variance of neuronal firing is critically controlled to preserve sensory sensitivity.

### (b) Choice probability for cylinder judgements is linked primarily to neurometric thresholds for cylinder disparity

The behavioural performance of the two monkeys in this task was typical of human psychophysical observers: the monkeys readily responded to the direction of rotation specified by the combination of motion and disparity within the stimulus, seemingly able to set aside the trial-by-trial changes in the orientation of the target. Those cortical neurons that preserve sensitivity to disparity when challenged in the same way may potentially be a single neuron correlate of the flexible and adaptive behaviour seen in psychophysical performance. We therefore turn to the question of whether the nervous system can make use of the information that is potentially available from these highly sensitive and versatile cortical neurons. We examined the association between neuronal firing and behavioural choice, quantified as CP (calculated over the full 2 s trial), in judgments of cylinder rotation for stimuli matched to the motion preference of the neuron or rotated away from the preferred–null motion axis (see figure 2*c* and *f* for example).

The mean choice probabilities were 0.64 for *Q*_pref_ (*n* = 93) and 0.62 for *Q*_non-pref_ (*n* = 68), similar to previously reported choice probabilities reported for the same task (0.67; Dodd *et al.* [6]). There was no significant difference in choice probabilities between cylinders with dot motion in the pref–null motion axis and those with sub-optimally aligned cylinders (*p >* 0.3, two-tailed *t*-test). In addition, there was no significant difference between the two monkeys, either for cylinders with dot motion aligned with the RF's preferred direction of motion or for sub-optimal dot motion in the cylinder stimulus (*p >* 0.3, two-tailed *t*-test).

The relationship between CP and direction of cylinder dot motion with respect to the RF preferences is presented graphically in figure 3*c*, analogous to figure 3*a* for neurometric sensitivity. There was no significant correlation (*r = –*0.11, *n* = 161, *p >* 0.15). Large changes in the visual match of the stimulus to the neuron's RF resulted in little change in the task-related CP signal found. In figure 3*c*, 61 out of the 161 neurons contributed two points, one each for the optimally and sub-optimally aligned stimuli. There was still no correlation, even if only one CP from each neuron was considered: either the set of CPs generated by the optimally orientated cylinder stimuli *Q*_pref_ (*r = –*0.042, *p >* 0.3) or the set generated by the sub-optimal stimuli *Q*_non-pref_ (*r = –*0.178, *p >* 0.3). The pattern of results for CP appears to be very similar to that for neurometric thresholds. This conclusion is reinforced by the observation that there is no correlation between CP and mean neuronal firing (*r = –*0.037*, n* *=* 161*, p >* 0.4) (figure 3*d*).

We also compared the simultaneously measured CP and neurometric threshold for cylinders with both optimally and sub-optimally aligned dot motion. With one exception [11], previous studies with single stimuli have shown a systematic tendency for more sensitive neurons to show higher choice probabilities [4,5,8,10,14,26]. Regardless of whether the dot motion of the cylinder was accurately matched to the neuronal preference or less well matched, there was a significant correlation (figure 3*e*) between CP and neurometric threshold (cylinders aligned to the preferred direction of motion: *r =* –0.332, *n =* 75, *p <* 0.01; cylinders with a sub-optimal motion axis: *r =* –0.456, *n =* 55, *p <* 0.001). Taking data from each monkey separately, the measured correlation was significant for Monkey F but not for Monkey R, most likely reflecting the limited data available for Monkey R. Overall, the present data confirm that the empirically observed relationship between CP and neuronal sensitivity holds for both optimally and sub-optimally matched stimuli.

### (c) For the same task, a single neuron's contribution to perceptual choice may vary between stimulus configurations

**Figure 4.**
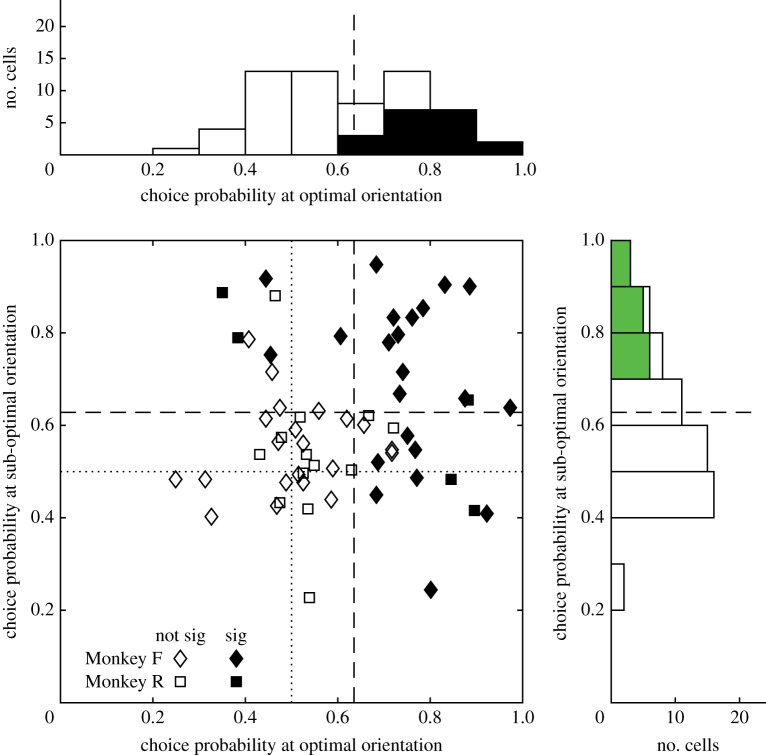
Choice probabilities (CPs) of single neurons during combined neuronal and behavioural recording with optimal and sub-optimal axis orientations of zero-disparity, ambiguous cylinders. The scatter plot shows results from 61 neurons from two monkeys, for cases where data were obtained at both optimal and sub-optimal cylinder axis orientations. The marginal histograms show the distribution of CPs at optimal cylinder orientation (upper) and sub-optimal orientation (right). Filled bars and symbols show CPs that are individually significant; open bars and symbols show non-significant values. The mean CP for the 61 paired results was 0.64 for the optimal orientation (0.65 monkey F, 0.60 monkey R) and 0.63 for the sub-optimal orientation (0.65 monkey F and 0.58 monkey R). All these mean values are significantly greater than 0.5, the value expected by chance (*t*-test, *p <* 0.0001). The mean CP for the optimally oriented cylinder (0.64) was greater than for the sub-optimally oriented cylinder (0.63), but this difference was not significant (*p >* 0.3, 2-tailed paired *t*-test and Wilcoxon signed rank test).

For those neuronal recordings that yielded measurable choice probabilities for both optimal and sub-optimally oriented cylinder stimuli with respect to the neuron's motion direction preference (*n* = 61), the pair of choice probabilities for ambiguous cylinders is plotted in figure 4. For both optimal and sub-optimally oriented stimuli, choice probabilities tended to be larger than 0.5. The filled symbols in figure 4 indicate neurons for which the CP was significantly different from 0.5 at either the optimal or the sub-optimal orientation of the cylinder axis. Choice probabilities below 0.5 would imply a consistent increase in neuronal firing to the ambiguous cylinder, contingent upon the monkey making a behavioural choice opposite to the neuron's stimulus preferences. Consistent with Dodd *et al.* [6], no neurons showed significant choice-related firing of this type. This was also the case for the enlarged datasets *Q*_pref_ and *Q*_non-pref_. Thus, significant choice probabilities were always greater than 0.5, consistent with the expectations derived from the stimulus preferences of the neuron, assessed separately for optimal and sub-optimal cylinder orientations.

The most obvious feature of the results in figure 4 is the similarity of the distributions of choice probabilities for the stimuli optimally and sub-optimally aligned with the neuron's motion preference. There is also an absence of any significant correlation between these two measures. Examination of figure 4 reveals cases where the neuron exhibits a strong CP for the stimulus not well matched to motion direction preference but a weak or non-existent CP for the optimally aligned stimulus. The general conclusion is also supported across the population of single neurons from the data plotted in figure 3*c–e* and especially the example neuron shown in figure 2*d*–*f*.

Since CP measures the association between neuronal firing and behavioural choice, this suggests that the neuronal population that is engaged when decisions are made about one orientation of the stimulus may differ from the population that is engaged for the other orientation.

### (d) No difference in the time course of choice probability for different stimulus configurations

Throughout this analysis, we have been concerned with searching for potential links between the decision-related activation of the neuron (assessed as CP) and the relationship of the stimulus to either the visual stimulus preference of the neuron or the task-specificity of the neuron. Although we have found thus far no evidence for a simple link between the stimulus preference of the neuron and its decision-related activity, we wondered whether stimuli that were less optimal in activating the neuron might have a weaker link to decision-related activity in some other way.

**Figure 5.**
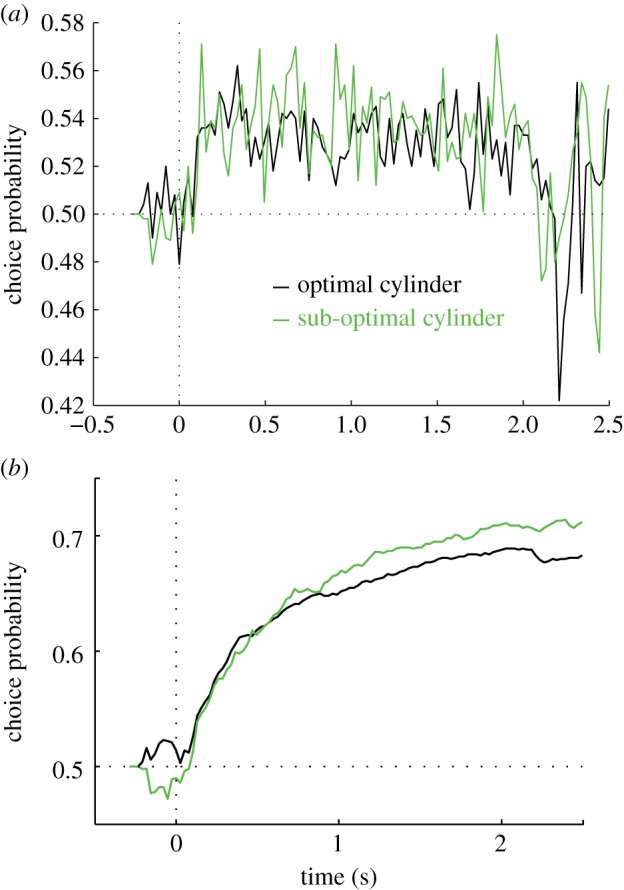
For the analysis of the time course of choice signals, we used neurons and cylinder configurations with a significant CP (permutation test, *p* < 0.05; cylinder optimally aligned to preferred motion direction: *n* = 23; sub-optimal dot motion: *n* = 15). (*a*) Instantaneous CP was calculated for each time point using data from a window with 41.7 ms duration. The timing and emergence of the CP signal did not differ when stimuli were less well matched to RFs and induced lower firing rates. (*b*) Same data as in (*a*), but CP was accumulated over the trial. Again, the time course of the emergence of the CP was similar for optimal cylinder stimuli that were aligned to the preferred–null direction of motion axis of the neuron (black) and for sub-optimal cylinders whose dot motion was less well matched (green).

We compared the emergence of CP measures over the course of the 2 s trial for cylinders that were aligned to the RF's direction preference and those that were not. If we take an instantaneous measure of CP, choice signals appear after around 100 ms, as previously found for the cylinder discrimination tasks [6]. They stay at a constant level throughout the remainder of the trial (figure 5*a*), which is consistent with the time course of related choice signals measured in V5/MT [5]. There is no difference whether the stimulus is well aligned to RF properties and elicits high firing rates or not. Similarly, when CP is calculated cumulatively, the two stimulus configurations yield similar CP trajectories (figure 5*b*). CP for cylinder disparity exhibits a similar time course, whether the stimulus is optimally matched to direction preference or not. Therefore, different levels of firing rate do not influence the emergence of a choice signal. There is no indication of any difference in the underlying neural mechanism that generates CPs in the two conditions.

## 4. Discussion

The problem of selecting groups or pools of neurons relevant for a specific perceptual task has been termed the ‘read-out’ problem [46]. For example, to provide neuronal signals for supporting a discrimination between leftwards and rightwards motion, the nervous system could assemble two broad pools of neurons, one whose members are primarily sensitive to leftwards motion and the other primarily sensitive to rightwards movement (or CW and CCW rotation for discrimination of cylinder rotation in our task). Then, a signal could be ‘read-out’ from these pools by taking the difference. Conceptually, the term ‘read-out’ is inherently unsatisfactory, as it implies a cognitive agent that can actively inspect the activity of a neuronal population and divide it into relevant pools. This terminology is therefore a temporary construct, convenient until a more satisfactory, neuron-based account can be put in place.

In this paper, we manipulate visual task sensitivity and the response level to the visual stimulus separately. We find that the spiking activity of sensory cortical neurons respects three important organizational principles. First, the neurometric sensitivity of single neurons is maintained in the face of substantial changes in firing rate. There is therefore useful information relevant to the performance of the visual task available from neurons in lower firing rate states, as well as those that are most strongly activated. For the rest of the brain to exploit this information effectively means that simple pooling models where membership of the pool is based on the absolute level of neuronal activation are only part of the overall picture.

Second, the previously observed relationship between CP and neurometric sensitivity is exhibited by single neurons at both high and low average firing rates for the individual neuron. Neurometric sensitivity is a measure of the potential relevance of the changes in firing rate of a recorded neuron for performance in the visual task. Thus, neurometric sensitivity is fundamentally a task-related property of a neuron and can only be defined with regard to the neuron's potential contribution to performance on a particular task. Recent experimental and modelling results have shown that the presence of a measurable CP for a neuron is not simply related to whether that neuron is ‘read-out’, that is to say, contributes to the decision-pool for the discrimination task [19,20,47]. Nonetheless, even if some neurons showing CP are not part of the decision pool, those models require that the activity of such neurons must still be correlated with neurons that are true members of the decision pool for read-out. Viewed in this way, a link between CP and the task-specific measure of neurometric sensitivity reflects the local architecture of connectivity within the network of cortical neurons. These local connections are fundamentally responsible for the correlated activity within the sensory neurons recorded there.

Third, taking CP as an indicator of the contribution of a neuron to a decision pool, we find that whether a neuron contributes to the pool in its high firing state is not predictive of whether it contributes in its low firing state. Hence, although neuronal sensitivity is very similar for the neuron in high and low firing states, CP is not. This observation is difficult to explain with a static set of local connections within the group of sensory neurons recorded in the cortical area. For if those local connections bring about CP by creating correlated activity between neurons that are truly in the read-out pool and additional neurons outside the pool, then a static set of connectivity would seem to predict that the local correlations (and hence CP) should not change with firing rate. The source of the elevation in firing rate that is the ‘choice signal’ may be bottom-up noise [17] or top-down feedback, related perhaps to attentional or reward signals [48–51].

We were able to disentangle neuronal firing rate, neurometric threshold and CP over a wider range of stimulus preferences than previously suggested [28], because we exploited the fact that perceptual decisions about cylinder rotation rely on V5/MT neurons that are selective for specific combinations of binocular disparity and direction of motion [3,6,7]. We studied the effect of neuronal mean firing rates—by manipulating a stimulus dimension (direction of motion) that was largely irrelevant to the stimulus dimension that formed the basis of the perceptual discrimination (binocular disparity).

In order to determine a neuronal pool in visual area V5/MT that is read-out for perceptual decisions in our stereo-motion task, we would also require a measure of interneuronal correlation, the amount of shared noise between neurons [17,27,52]. It is the interaction between shared noise and CP that can help define the size of the decision pool and whether it is read out optimally ([17,19–21], but see also [53] for choice probabilities in the LGN without interneuronal correlations). It will be important to establish the level of interneuronal correlation for our task in V5/MT. However, some conclusions about the nature of the perceptual decision pools in our stereo-motion task can be made based on the distribution of choice probabilities we found.

Previous research has suggested that the pool of potential neurons carrying choice probabilities for a specific task is large [10,28], but also that neurons can carry choice signals that do not necessarily contribute directly to perception but represent shared signals, for example cognitive feedback signals [47,54]. So, the pool of sensory neurons that show significant choice probabilities comprises some that contribute to perception [24], but also other neurons directly or indirectly connected to those with perceptual signals. We would expect that potentially many task-relevant neurons, including those that contribute directly to perception, show large choice probabilities; some simply based on the pool of neurons they are connected to. The surprising aspect of our results is the absence of choice signals for specific stimulus configurations when a very similar stimulus for the same task shows a large CP and this in a fully interleaved experimental design.

Our experimental findings are well explained by a model of decision pools called the ‘micropool model’ because the relevant neurons are assembled into several sets of small-sized pools [55]. The neurons within a pool share similarities of stimulus preferences, although they may differ considerably in the sensitivity of individual neurons to the task. The neurons within the micropool share cortical connections, thus leading to measurable interneuronal correlations between the firing patterns of neurons within the micropool. Recruitment to the decision process is at the level of the micropool: that is to say, if a micropool is recruited to the decision pool, then all of the neurons within the micropool then make a weighted contribution to the decision. Different pools, which can comprise neurons with broadly comparable sensitivity for a given task, could flexibly contribute to perceptual choices about different configurations of a stimulus as in this paper. Thus, there are potentially different neuronal strategies available to solve a given perceptual task—such as using direction-selective V5/MT neurons that are not disparity-selective for a motion discrimination task or neurons that are both direction- and disparity-selective [46]. Such micropools would also provide the flexibility to be recruited to different representations of category boundaries, like ‘left’ and ‘right’ or ‘CW’ and ‘CCW’ in downstream sensori-motor areas [56]. Association of micropools and perceptual decision task might be modified during development and perceptual learning.

## 5. Conclusion

The neuronal pool carrying perceptual decision signals comprises a specific, local subset of neurons sensitive to the task. When a stimulus parameter that is irrelevant to the perceptual task is altered, a neuron's neurometric sensitivity for the task can be maintained over a wide range of response levels. However, a neuron might not carry perceptual decision signals for all versions of a visual stimulus, even when the task remains unchanged.

## Data accessibility

Data are available on request from the corresponding author.
